# JAK2 V617F detected in two B-cell chronic lymphocytic leukemia patients without coexisting Philadelphia chromosome-negative myeloproliferative neoplasms: A report of two cases

**DOI:** 10.3892/ol.2014.2168

**Published:** 2014-05-22

**Authors:** YI-NING YANG, YOU-WEN QIN, CHUN WANG

**Affiliations:** Department of Hematology, Shanghai First People’s Hospital, Medical College, Shanghai Jiao Tong University, Shanghai 200080, P.R. China

**Keywords:** JAK2 V617F, B-cell lymphocytic leukemia, Philadelphia chromosome-negative myeloproliferative neoplasm

## Abstract

The JAK2 V617F mutation has been observed in patients with Philadelphia chromosome-negative myeloproliferative neoplasms (Ph-MPNs), including polycythemia vera, essential thrombocythemia and idiopathic myelofibrosis. This mutation has also been observed in a small number of other myeloid malignancies, such as acute myeloid leukemia, chronic myeloid leukemia and myelodysplastic syndrome. The JAK2 V617F allele has rarely been evaluated in lymphoproliferative disorders. In total, 28 JAK2 V617F-positive B-cell lymphocytic leukemia (B-CLL) patients have previously been reported and all presented with Ph-MPN concomitantly. However, following investigation of the JAK2 V617F mutation in 63 B-CLL patients at the Shanghai First People’s Hospital (Shanghai, China) between January 2008 and December 2012 via allele-specific polymerase chain reaction, two B-CLL patients without a history of Ph-MPN were identified to carry the JAK2 V617F allele.

## Introduction

A somatic point mutation in the JAK2 gene, 1849G>T, which changes amino acid residue 617 of the kinase from valine to phenylalanine (termed JAK2 V617F), has been identified by various studies in a substantial number of Philadelphia chromosome-negative myeloproliferative neoplasm (Ph-MPN) patients (1). This mutation has also been identified in a small number of other myeloid malignancies (2,3), however, rarely presents in lymphoid malignancies (4,5). Following a search of the English language literature between 2006 and January 2013 using the search terms ‘B-cell lymphocytic leukemia’ (B-CLL) and ‘JAK2’, 28 JAK2 V617F-positive B-CLL patients were identified (6–15). All of these patients exhibited a Ph-MPN concomitantly. The coexistence of two chronic myeloid and lymphoid neoplasms in a patient raises the possibility that the neoplasms are derived from the same pluripotent stem cell, however, they may be purely coincidental. This led to the retrospective analysis of the JAK2 V617F mutation in 63 B-CLL patients that were diagnosed at the Department of Hematology (Shanghai First People’s Hospital, Shanghai, China) between January 2008 and December 2012. Two B-CLL patients were identified to carry the JAK2 V617F allele. Notably, these two patients did not have a history of Ph-MPN, which is not consistent with the previously reported cases. Patients provided written informed consent.

## Case reports

### Case 1

**Figure 1:**
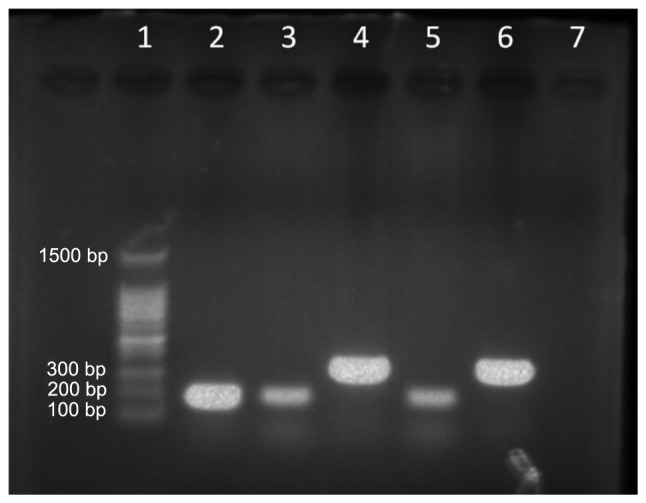
JAK2 V617F mutation detected by allele-specific polymerase chain reaction. Lanes; 1, molecular weight marker (100–1,500 bp); 2, JAK2 V617F-positive control (203 bp); 3, JAK2 V617F mutation allele (case 1); 4, wild-type allele for JAK2 (364 bp, case 1); 5, JAK2 V617F mutation allele (case 2); 6, wild-type allele for JAK2 (364 bp, case 2); and 7, negative control.

A 57-year-old male patient was admitted to the Department of Hematology (Shanghai First People’s Hospital, Shanghai, China) with leukocytosis in September 2006. The patient’s whole blood count (WBC) was elevated (14.5×10^9^/l; reference range, 3.97–9.15×10^9^/l), while the hemoglobin (Hb) and platelet (PLT) counts were within the reference ranges [14.7 g/dl (reference range, 13.1–17.2 g/dl) and 231×10^9^/l (reference range, 85–303×10^9^/l), respectively]. The circulating lymphocyte percentage was 49%. In the bone marrow (BM), the cellularity and lymphocyte compartment size were increased. Clinical examination revealed an enlarged spleen and flow cytometric analysis of the patient’s peripheral blood (PB) exhibited the B-CLL phenotype. The total lymphocyte count was 7.1×10^9^/l and gated CD45^+^ cells showed positivity for cluster of differentiation (CD)19 (88.4%), CD20 (39%), CD5 (65.9%), CD41 (16.9%) and HLA-DR (93.2%). The patient was negative for CD38 (5.1%) and ZAP70 (2.6%). The diagnosis of stage II B-CLL was determined according to the Rai classification (16). The patient was followed up for four years with fludarabine (50 mg/m^2^ on days one to five) treatment. Blood examination showed that the WBC was between 6.3 and 40×10^9^/l, the lymphocyte percentage was between 45 and 79%, the Hb level was between 139 and 149 g/l and the PLT count was between 161 and 231×10^9^/l. In October 2010, cytogenetic analysis revealed a normal karyotype and the JAK2 V617F mutation was detected by allele-specific polymerase chain reaction (AS-PCR; Fig. 1) (1). The patient did not exhibit any identifiable signs, symptoms or laboratory findings for Ph-MPN. In April 2011, the patient was diagnosed with advanced carcinoma of the gallbladder during the course of B-CLL and succumbed to the disease in December 2012.

### Case 2

A 63-year-old female patient was admitted to the Department of Hematology (Shanghai First People’s Hospital) with leukocytosis in November 2010. The patient’s WBC was elevated (98.6×10^9^/l; reference range, 3.69–9.16×10^9^/l), whilst the Hb and PLT counts were within the reference ranges [11.5 g/dl (reference range, 11.3–15.1 g/dl) and 101×10^9^/l (reference range, 85–303×10^9^/l), respectively]. The circulating lymphocyte percentage was 91%. The PB smear demonstrated an absolute lymphocytosis of predominantly small, mature lymphocytes. The BM showed an increased number of lymphocyte compartments and splenomegaly was present on the abdominal ultrasound. Flow cytometric analysis of the patient’s PB exhibited the B-CLL phenotype. The total lymphocyte count was 89.7×10^9^/l and the gated CD45^+^ cells showed positivity for CD19 (77.1%), CD20 (77.7%), CD13 (11.7%), CD5 (7.4%), CD2 (8.8%), CD4 (7.6%), CD10 (7.6%), CD22 (1.4%), CD14 (1.6%), CD15 (6.4%), CD33 (2.5%), CD38 (3.6%), CD41 (6.07%) and CD7 (7.7%). Cytogenetic analysis of the BM revealed a normal karyotype and the JAK2 V617F mutation was detected by AS-PCR (Fig. 1). The typical characteristics of B-CLL, such as trisomy 12 and the deletion of 11q22.3, 13q14 and 17p13, were not detectable by fluorescent *in situ* hybridization. The diagnosis of stage II B-CLL according to the Rai classification was determined. The patient was treated with rituximab (500 mg/m^2^ on day one) plus fludarabine (35 mg/m^2^ on days two and three). Owing to the complete remission status over the following 1.5 years, the patient returned to the Xinyang Sixth People’s Hospital (Henan, China) in March 2012.

## Discussion

Of the 28 cases of JAK2 V617F-positive B-CLL patients reported in the literature (Table 1) (6–15), the male and female ratio was 1.6:1 (17 males vs. 11 females), the median age of the males was 69 years (range, 55–94 years) and was 74 years (range, 58–82 years) for the females. In total, 27 patients exhibited coexistent Ph-MPN (essential thrombocythemia, n=16; polycythemia vera, n=10; and idiopathic myelofibrosis, n=1). The remaining B-CLL patient reported by Musolino *et al* (13) was without detailed clinical data. The current study presents two younger JAK2 V617F-positive B-CLL patients without any history of Ph-MPN; the JAK2 V617F allele was detected in one patient after the fourth year of follow-up and the other was a newly diagnosed B-CLL patient.

In order to understand why the JAK2 V617F mutation existed in B-CLL patients it is necessary to determine whether the JAK2 V617F mutation exists in lymphocytes. The JAK2 V617F mutation in Ph-MPN patients was hypothesized to be present in stem, myeloid and erythroid cells rather than in lymphocytes (17). Previous studies (18–21) identified the JAK2 V617F mutation in B and T lymphocytes, as well as in natural killer cells in Ph-MPN patients. However, this remains controversial in JAK2 V617F-positive CLL patients. This mutation has been identified in B or T cells by various studies (6–8), while other studies have drawn contrasting conclusions (11–15). As the DNA samples used in the current patients had been stored, identification of the JAK2 V617F mutation in the lymphoid compartment using cell sorting was not possible. However, it was agreed that JAK2 V617F may exist in the lymphoid and myeloid cells, which are involved in the progress of B-CLL.

The role of JAK2 V617F in the pathogenic mechanism of B-CLL requires investigation. The V617F substitution induces a conformational shift that alleviates repressive interactions between its JH1 and JH2 domains, resulting in the constitutive activation of JAK2 (22,23), which enhances downstream signaling pathways, such as Janus kinase (JAK)-signal transducers and activators of transcription (STAT) and leads to the proliferation of cells in Ph-MPN (24). Furthermore, the activation of the JAK-STAT signaling pathway has been documented in lymphoid malignancies (25–28). Thus, it is reasonable to propose that JAK2 V617F mutations lead to the constitutive activation of the JAK-STAT signaling pathway in lymphocytes, subsequently resulting in cellular proliferation in the absence of normal cytokine stimulation. This may lead to increased cell numbers and indicate a novel mechanism that results in B-CLL.

Notably, the two JAK2 V617F-positive B-CLL patients described in the current study were without a Ph-MPN. One explanation for this is that the JAK2 V617F mutation alone is not sufficient to induce a Ph-MPN, as it may occasionally be found in hematologically normal individuals. Sidon *et al* (29), reported that the JAK2 V617F mutation is detectable at low levels in ~10% of the PB of healthy donors. A larger study of 3,700 individuals in Chinese hospitals revealed the presence of JAK2 V617F in 1% of the normal population (30). An additional explanation is that the JAK2 V617F mutation only represents an early molecular event, which precedes clinical and hematologic abnormalities. Certain patients may never reach the full-scale MPN phenotype prior to succumbing to other diseases. Due to mortality and loss to follow-up in the patients included in the current study, it was impossible to determine whether the patients later developed Ph-MPNs.

In conclusion, the current study presents two B-CLL patients with the JAK2 V617F mutation. Compared with patients in previous reports (6–15), the present patients did not exhibit the Ph-MPN phenotype. Although JAK2 V617F existence in B-CLL is rare, clinicians must be aware that it is a possibility. By comparing the previous and current cases, the existence of JAK2 V617F in lymphocytes was reviewed and a novel mechanism that results in B-CLL was proposed. In order to support these views, further larger studies regarding JAK2 V617F-positive B-CLL are required.

## Figures and Tables

**Table I:** Cases of JAK2 V617F reported in B-cell CLL patients.

Year	First author (ref.)	Age, years/Gender	Initial diagnosis	Clinical disease process
2006	Hussein *et al* (15)	79/M	PV	PV to CLL
2007	Henry *et al* (14)	58/F	ET	ET to CLL
2009	Tabaczewski *et al* (9)	72/M	CLL and ET	CLL and ET
		82/M	CLL and ET	CLL and ET
2009	Kodali *et al* (8)	80/M	CLL and ET	CLL and ET
2009	Musolino *et al* (13)	72/F	ET	ET to CLL
		57/M	CLL	NA
		68/F	CLL and ET	CLL and ET
		78/F	ET	ET to CLL
		74/F	CLL	CLL to ET
		67/M	ET	ET to CLL
		74/M	CLL	CLL to ET
		69/M	PV	PV to CLL
2011	Laurenti *et al* (10)	73/F	CLL and PV	CLL and PV
		82/F	ET	ET to CLL
		76/M	PV	PV to CLL
		80/F	ET	ET to CLL
		55/M	CLL	CLL to ET
		79/M	CLL	CLL to ET
		77/M	CLL	CLL to PV
		69/M	PMF	PMF to CLL
2012	Stijnis *et al* (6)	60/M	PV	PV to CLL
		60/M	PV	PV to CLL
2012	Wei *et al* (11)	94/M	CLL	CLL to ET
2012	Eskazan *et al* (7)	56/M	CLL and ET	CLL and ET
2013	Swierczek *et al* (12)	79/F	PV	PV to CLL
		67/F	PV	PV to CLL
		78/F	CLL and PV	CLL and PV
2013	Current report	57/M	CLL	CLL
		63/F	CLL	CLL

M, male; F, female; PV, polycythemia vera; ET, essential thrombocythemia; CLL, chronic lymphocytic leukemia; NA, not applicable; PMF, primary myelofibrosis.
